# Measuring Resilience in Adult Women Using the 10-Items Connor-Davidson Resilience Scale (CD-RISC). Role of Trauma Exposure and Anxiety Disorders

**DOI:** 10.1371/journal.pone.0039879

**Published:** 2012-06-29

**Authors:** Jacqueline Scali, Catherine Gandubert, Karen Ritchie, Maryvonne Soulier, Marie-Laure Ancelin, Isabelle Chaudieu

**Affiliations:** 1 Inserm, U1061, Montpellier, France; 2 University of Montpellier 1, Montpellier, France; 3 School of Public Health, Imperial College, London, United Kingdom; 4 CRLC Val d'Aurelle-Paul Lamarque, Montpellier, France; Wayne State University, United States of America

## Abstract

**Purpose:**

Resilience is the ability of individuals to adapt positively in the face of trauma. Little is known, however, about lifetime factors affecting resilience.

**Methods:**

We assessed the effects of psychiatric disorder and lifetime trauma history on the resilience self-evaluation using the Connor-Davidson Resilience Scale (CD-RISC-10) in a high-risk-women sample. Two hundred and thirty eight community-dwelling women, including 122 participants in a study of breast cancer survivors and 116 participants without previous history of cancer completed the CD-RISC-10. Lifetime psychiatric symptoms were assessed retrospectively using two standardized psychiatric examinations (Mini International Neuropsychiatric Interview and Watson's Post-Traumatic Stress Disorder Inventory).

**Results:**

Multivariate logistic regression adjusted for age, education, trauma history, cancer, current psychiatric diagnoses, and psychoactive treatment indicated a negative association between current psychiatric disorder and high resilience compared to low resilience level (OR = 0.44, 95% CI [0.21–0.93]). This was related to anxiety and not mood disorder. A positive and independent association with a trauma history was also observed (OR = 3.18, 95% CI [1.44–7.01]).

**Conclusion:**

Self-evaluation of resilience is influenced by both current anxiety disorder and trauma history. The independent positive association between resilience and trauma exposure may indicate a “vaccination” effect. This finding need to be taken into account in future studies evaluating resilience in general or clinical populations.

## Introduction

The concept of resilience has been defined as the capacity of individuals to cope with traumatic events, namely the capacity to “maintain relatively stable, healthy levels of psychological and physical functioning (…) as well as the capacity for generative experiences and positive emotions” [1]. While some authors have argued that resilience cannot be directly measured but only inferred from the study of both risk factors and positive adaptation following an adverse life event, [2], [3], others have proposed quantification of resilience using specific scales [4]. Among the scales developed to explore resilience in adults, two types of instruments have been used. The first one measures a subject's self-evaluation of prior experience in successfully overcoming stressful events and positive changes. In this case the resilience evaluation requires the presence of a stressor or a research participant’s recollection of their response to a previous one. The second group measures subjective factors, which are considered as determinants of resilience (e.g. personal competence or social resources) and may prospectively determine resilience but does not evaluate resilience itself [5].

Of the first group instruments measuring resilience, the Connor-Davidson Resilience Scale (CD-RISC) is a self-administered scale of 25 items that exhibits good psychometric properties [6]. It was designed to be widely applicable to different populations establishing norms for resilience in normal and clinical samples, and to assess the extent to which resilience scores can change in response to treatment [6]. CD-RISC was initially considered to be multidimensional, with five factors corresponding to personal competence/tenacity, trust in one's instincts/tolerance of negative affect, positive acceptance of change/secure relationships, control, and spirituality [6]. However further studies across independent samples of different ages and cultures has revealed instability in the factor structure [7], [8], [9], [10] leading to the validation of an abridged 10-items version, the CD-RISC-10. The retained 10 items reflect the ability to bounce back from the variety of challenges that can arise in life [11]. This unidimensional version has equally excellent psychometric properties as the longer version, is applicable for different cultures and is quite adapted to large epidemiological studies [11], [12], [13].

In a sample of 132 students, Campbell-Sills et al. have shown that regardless the CD-RISC version (complete or abridged) resilience was associated with personality dimensions such as neuroticism or extraversion as well as coping styles [11], [14]. The main relevance of these studies is that authors have attempted “to capture the essence of resilience” showing that resilience scores could moderate the relationship between childhood emotional neglect and current psychiatric symptoms [11], [14]. More recently a similar observation was reproduced in a highly traumatized, at risk, urban population (median age 36 years, predominantly African American). The authors showed that childhood abuse or later traumas of adult life contributed to current depressive symptoms severity while resilience mitigated it [15]. In all these cross-sectional studies, the authors have implicitly considered resilience as a personality trait, assuming that high resilience score lead to fewer psychiatric symptoms in individuals. It is however, also conceivable that psychiatric symptoms can cause these persons to evaluate themselves as less resilient [14]. Thus the nature of the relationship between resilience score and current psychiatric symptoms in adults remains to be specified. Likewise the impact of past psychiatric diagnoses on resilience score is largely unknown. In addition research undertaken in general populations with the CD-RISC is based on the assumption that resilience is observed independently of the level of the stress exposure. Past traumatic events may affect the development of post-traumatic symptoms following an adverse life event and thus positive adaptation/resilience [16]. However the impact of previous trauma on self-evaluation of resilience in the face of current moderate levels of stress is largely unknown.

Given the increasing interest of psychiatric research in the relative capacity for healthy adaptation to life adversities as well as the clinical relevance of resilience measure [6], this retrospective epidemiological study aims to evaluate resilience in a high-risk women sample, using the abridged version of the CD-RISC, taking into account life-time history of trauma (distinguishing personal from non-personal events), socio-demographic characteristics and lifetime mental health.

## Methods

### Ethics statement

Ethics approval for the study was given by the national ethics committee of the National Institute of Health and Medical Research (Inserm, France). Written informed consent was obtained from all participants involved in the study.

### Participants

The data were derived from a comparative study of breast cancer survivors and women without previous history of cancer in which we have previously observed a contrasted pattern of current psychiatric disorder [17].

Briefly, women were recruited between November 2002 and April 2004 in waiting rooms of specialist breast radiologists as well as in the Regional Cancer Hospital Val d'Aurelle-Paul Lamarque in Montpellier, France. The inclusion conditions were being aged from 18 to 75 years and having a mammography. The women in the cancer group had received a primary breast cancer diagnosis (stage I–III [18]) one to three years before the interview and were in remission but with no active treatment (except for hormonal treatment). All the women were interviewed after their mammogram was taken. The standardized interview included resilience and mental health measures as well as questions on socio-demographic, lifestyle characteristics and current medications. Psychoactive treatment consisted of antidepressant and anxiolytic medications. Of the 324 participants, only women with a complete psychiatric evaluation and no missing data for the variables considered in the analysis were included. The present analysis was thus conducted on 238 participants (122 exposed to breast cancer and 116 non-exposed). These women did not differ from those excluded from the analysis with regard to age (p = 0.10), marital status (p = 0.10) and education (p = 0.22).

### Resilience measure

The original CD-RISC is a 25-item scale assessing resilience during the last month, with higher scores indicating higher resilience capacity. Each item is rated on a 5-point range of responses from not true at all (0) to true nearly all time (4). The total score ranges from 0–100. A preliminary study of its psychometric properties in general population and patient samples showed adequate internal consistency, test-retest reliability, and convergent and divergent validity [6]. The abridged CD-RISC-10 version reflects the ability to tolerate experiences such as change, personal problems, illness, pressure, failure and painful feeling (item's examples: “Able to adapt to change”, “Tend to bounce back after illness or hardship”, and “Can stay focused under pressure”). In our sample, the CD-RISC-10 showed high internal consistency (Cronbach's α = 0.88). Due to non normal distribution, total scores were categorized into tertiles to examine possible non-linear association.

### Mental health measures

The Watson's PTSD Interview (PTSD-I, DSM-IIIR; internal consistency, α = 0.92 and test-retest reliability total score = 0.95) [19] was used to obtain both lifetime and current diagnoses of post-traumatic stress disorder (PTSD), using the validated French hetero-questionnaire version [20], [21]. The first question identifies past traumatic events spontaneously evoked by the participants. At this step the nurse specified the question listing a large number of traumatic events. The second question, concerning the most frightening personal experience in the past, was only asked if there was no response to the first question. If this experience is a traumatic event as defined according to DSM-IV criteria, the questionnaire is continued focusing on the most traumatic event. This questionnaire thus lists all past traumatic events declared by the participants, including cancer when reported as such. The last part of the PTSD-I then includes 17 items corresponding to specific DSM-IIIR symptoms. Participants answer each question using a 7-point Likert scale ranging from “1 (never)” to “7 (extremely)”, a score of “4 = commonly” being considered to be sufficient to meet the relevant DSM symptom criterion. The main advantage of PTSD-I is to provide continuous measures of the severity of the disorder for every symptom. This assessment tool also allows measuring partial PTSD, defined as endorsing symptoms sufficient to meet criteria for two of three PTSD symptom clusters [22].

A validated standardized psychiatric interview, the Mini International Neuropsychiatric Interview (MINI; DSM-IV criteria; French version 5.00) was used to investigate dysthymia and lifetime major depressive disorder (MDD), mania, and anxiety disorders, *e.g.* phobia, general anxiety disorder (GAD), obsessive-compulsive disorder, panic disorder with and without agoraphobia [23]. Case-level of current mood disorder was defined as a MINI diagnosis of current MDD, or current mania or current dysthymic disorder. This interview was administered by the same research nurse trained by a psychiatrist.

The General Health Questionnaire 28 (GHQ-28) is a self-administered screening test designed to detect current non-psychotic psychiatric disorder in community settings [24]. Participants were asked to assess their state in recent weeks compared to their usual state. This scale consists of four sub-scales for somatic symptoms, anxiety and insomnia, social dysfunction and severe depression. It comprises seven positive and 21 negative items with a total score ranging from 0 to 28 (high level of current disorder). In our sample the median score, was chosen as a cut-off.

### Statistical analysis

Comparisons of the socio-demographic variables between groups were carried out using the Chi-square test for categorical variables and Wilcoxon's test for quantitative variables. Due to non normal distribution of the resilience scores in our women sample, multinomial logistic regression models were used to study the association between CD-RISC-10 scores categorized as tertile groups with low (reference = R1), intermediate (R2) and high (R3) resilience level-and current and past mental health, or life-time serious event exposure. A multivariate logistic regression included covariates that were commonly reported in the literature (age, education level) [25], [26] or found to be associated with the level of resilience in our sample at p<0.10 (history of lifetime trauma and breast cancer, current psychiatric disorder, and psychoactive treatment). SAS version 9.1 was used for the statistical analyses with a significance level of p<0.05 (SAS Institute, Inc., North Carolina).

## Results

### Resilience according to sample characteristics

In this female sample the median score (Q25–Q75) on the CD-RISC-10 was 27 (range 22–32). Marital status, age and education level were not significantly associated with resilience level (Table 1).

**Table 1:** Association between socio-demographic characteristics and resilience level.

CD-RISC-10 score	R1 (n = 78)	R2 (n = 76)	R3 (n = 84)	
Variable	%	%	%	p global
Marital status				
Single/widowed/separated	23.08	23.68	23.81	.99
Married/cohabiting	76.92	76.32	76.19	
Education > 9years	50.00	52.63	35.71	.07
Median age (Q25-Q75)	53 (46–62)	54 (49–59)	52 (46–60)	.68

Note: Resilience scores are classified in three categories: CD-RISC score ≤23 (R1),23< CD-RISC ≤29(R2) and CD-RISC score >29 (R3).

Table 2 shows resilience levels as a function of lifetime psychiatric health and history of exposure to a serious traumatic event. Women scoring high on the CD-RISC-10 (group R3) tended having lower risk of current psychiatric disorder (p = 0.07). Higher resilience was associated with less anxiety disorder (p = 0.02), notably GAD (p = 0.04). There was no significant association between resilience level and current mood disorders including MDD. A similar pattern was observed for women showing an intermediate resilience level (group R2) except that they were also at lower risk of current mood disorder (p = 0.05). No significant differences were observed between R2 and R3 groups. Finally, no significant association was observed between resilience levels and past psychiatric diagnoses.

**Table 2:** Association of Lifetime Psychiatric Diagnoses and Lifetime Serious Event Exposure, with Resilience Level.

CD-RISC-10 score	R1 (n = 78)	R2 (n = 76)	R3 (n = 84)					
Variable	%	%	%	P global	OR [95% CI] (R2 vs.R1)	p	OR [95% CI] (R3 vs.R1)	p
***Current diagnosis***								
At least 1 psy.disorder.	42.31	23.68	28.57	.06	0.42 [0.21;0.85]	.02	0.55 [0.28;1.05]	.07
At least 1 mood disorder^a^	23.08	10.67	19.05	.51	0.40 [0.16;0.98]	.05	0.79 [0.37;1.67]	.53
At least 1 anxious disorder	33.33	21.05	16.67	.01	0.53 [0.26;1.10]	.09	0.40 [0.19;0.84]	.02
MDD	15.38	7.89	11.90	.49	0.47 [0.17;1.33]	.16	0.74 [0.30;1.83]	.52
GAD	23.08	10.53	10.71	.02	0.39 [0.16;0.97]	.04	0.40 [0.17;0.95]	.04
Full or partial PTSD	7.69	11.84	5.95	.69	1.61 [0.54;4.77]	.39	0.76 [0.22;2.60]	.66
High GHQ28 score^c^	58.33	47.22	51.90	.46	0.64 [0.33;1.24]	.18	0.77 [0.41;1.47]	.43
Psychoactive drug use	35.90	34.21	23.81	.10	0.93 [0.48;1.80]	.83	0.56 [0.28;1.11]	.09
***Past diagnosis*** b								
At least 1 psy. disorder	25.64	36.84	30.95	.50	1.69 [0.85;3.37]	.14	1.30 [0.65;2.59]	.45
At least 1 anxious disorder.	11.54	17.11	20.24	.14	1.58 [0.63;3.95]	.33	1.94 [0.81;4.66]	.14
MDD	30.77	40.79	29.76	.86	1.55 [0.80;3.01]	.20	0.95 [0.49;1.87]	.89
GAD	14.10	15.79	9.52	.39	1.14 [0.47;2.77]	.77	0.64 [0.24;1.69]	.37
***Serious event history***								
Trauma	23.08	38.16	40.48	.02	2.06 [1.02;4.15]	.04	2.27 [1.14;4.49]	.02
Breast cancer event^d^	23.08	32.89	26.19	.10	2.65[1.20;5.88]	.02	1.83[0.84;4.02]	.13

Note: Resilience scores are classified in three categories: CD-RISC score ≤23 (R1), 23< CD-RISC ≤29 (R2) and CD-RISC score >29 (R3).

a Current mood disorder corresponds to participants who fulfilled criteria for MDD, mania and dysthymic disorder.

b Free of current psychiatric disorder.

c High GHQ28 score corresponds to score ≥median.

d The analysis was carried out only on the subgroup of women with an history of early breast cancer but no history of traumatic event (n = 65).

Regarding lifetime serious events evaluated using the PTSD-I, two groups were successively considered in the analysis i) “having experienced a traumatic event” (any type; n = 81) and ii) “having experienced an early breast cancer but no trauma” (n = 65). The most common traumatic events in the first group were the sudden unexpected death of a close one (n = 35; 4 of whom were aged 12 years or younger and 3 were aged 13 to 18 years) and the cancer disease (n = 30) (Table 3). In the subsample of cancer survivors (n = 122), 30 women reported the cancer disease as a traumatic event and 27 reported another traumatic event, whereas in the subsample of women without a history of cancer disease (n = 116), 24 women reported a lifetime traumatic event.

**Table 3:** Frequencies of Trauma Categories by Resilience Level in Women who have been exposed to a Lifetime Traumatic Event (n = 81).

CD-RISC-10 score	R1 (n = 18)	R2 (n = 29)	R3 (n = 34)
Trauma category	n (%)	n (%)	n (%)
***Non-assaultive and personal***			
Cancer disease	11 (36.7%)	8 (26.7%)	11 (36.7%)
Other life-threatening illness (except cancer)	0	2	0
Natural disaster	0	2	0
Discovering a dead body	0	0	1
***Non-assaultive and non-personal***			
Sudden, unexpected death of a close one	4 (11.4%)	14 (40.0%)	17 (48.6%)
Serious accident or life-threatening illness of a close one	1	0	3
***Assaultive***	1	2	1
***Enable to cite the traumatic event***	1	1	1

Note: Resilience scores are classified in three categories: CD-RISC score ≤23 (R1), 23< CD-RISC ≤29 (R2) and CD-RISC score >29 (R3).

Compared to women with low resilience levels, women scoring at an intermediary level were significantly more likely to have been exposed to recent breast cancer or a lifetime traumatic event by more than 2-fold. A similar pattern was observed for the women scoring at a high resilience level, although the association with a history of breast cancer failed to be significant (p = 0.13) (Table 2).

**Figure 1:**
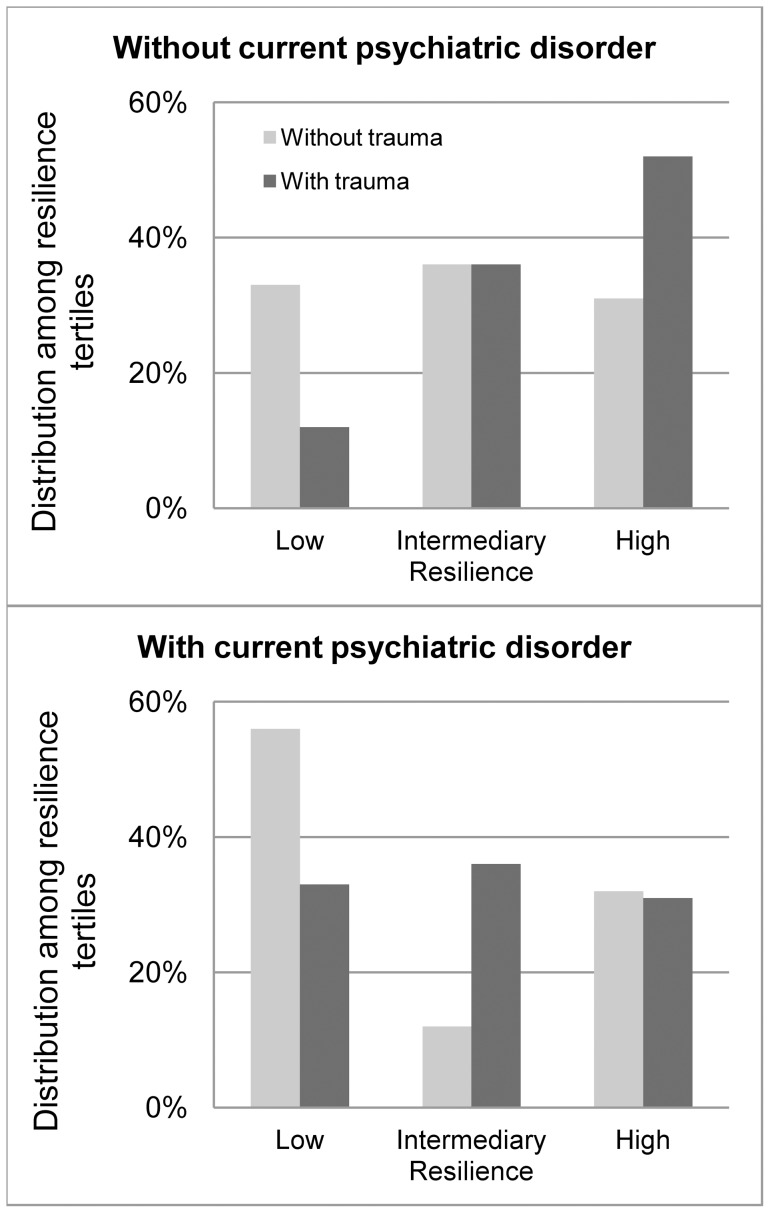
Women's resilience as a function of lifetime trauma and current psychiatric disorder.

Figure 1 shows the distribution of women among resilience groups as a function of lifetime trauma and current psychiatric disorder. The women without psychiatric disorder and reporting no lifetime trauma were distributed equally among the three groups, whereas those having experienced a trauma were more frequently distributed in the high than in the low resilience group. For women with current psychiatric disorder an inverse pattern was observed, only women without lifetime trauma distributed unequally among groups, being more frequently in the low resilience group.

### Multivariate analyses of factors associated with resilience

In multi-adjusted model current psychiatric disorder and past trauma remained significantly and independently associated with resilience level (Table 4). Women with intermediary and high resilience levels were more likely to report a lifetime trauma (OR = 2.38 and 3.18, respectively). History of breast cancer was also associated with resilience for the intermediary group although this failed to be significant in the high resilience group. Current psychiatric disorders were 2.3 and 3.3 less frequent in the groups with high and intermediary resilience levels, respectively.

**Table 4:** Multivariate Logistic Regression Analyses of Factors associated with the Level of Resilience.

Variable	OR [95% CI] (R2 vs.R1)	p	OR [95% CI] (R3 vs.R1)	p
Age	1.00 [0.96; 1.03]	.92	0.98 [0.95; 1.02]	.33
Education>9 years	1.12 [0.56; 2.23]	.75	0.47 [0.24; 0.94]	.03
History of lifetime traumatic event	2.38 [1.07; 5.32]	.03	3.18 [1.44; 7.01]	.004
History of breast cancer	2.03 [1.00; 4.09]	.05	1.53 [0.77; 3.06]	.23
At least 1 current psychiatric disorder	0.30 [0.14; 0.67]	.003	0.44 [0.21; 0.93]	.03
Psychoactive treatment	0.99 [0.47; 2.11]	.99	0.51 [0.24; 1.10]	.09

Resilience scores are classified in three categories: CD-RISC score ≤23 (R1), 23< CD-RISC ≤29 (R2) and CD-RISC score >29 (R3).

Among the 75 women with current psychiatric disorder, 32 have pure anxiety disorder, 19 pure mood disorder and 24 comorbid anxiety and mood disorder. Women with high resilience compared to those with low resilience reported 5-fold less current anxious and depressive comorbidity (OR = 0.21, 95%CI [0.06–0.73], p = 0.01) and tended to be at 3-fold lower risk of pure current anxious disorder (OR = 0.37, 95%CI [0.13–1.05], p = 0.06) whereas they did not differ significantly regarding pure mood disorder (OR = 1.01, 95%CI [0.32–3.21], p = 0.99).

## Discussion

In this sample of adult women the level of resilience measured with the CD-RISC-10 was negatively associated with the presence of current psychiatric disorder and positively and independently associated with previous history of trauma.

### Resilience and mental health

Resilience and related concepts such as “hardiness” have been reported as indices of mental health [27]. The negative association between current psychiatric diagnoses and resilience has been initially described using CD-RISC-25 [6]. Lower resilience scores were reported in psychiatric outpatients and in GAD patients compared to the general population. In PTSD patients, a greater global clinical improvement after pharmacologic treatment was associated with a greater increase in CD-RISC resilience scores [28]. Our data confirm and extend these findings in non-psychiatric patients using CD-RISC-10. We did not find a significant association between past psychiatric diagnoses and resilience levels, suggesting that global resilience scores could be a reversible state-like index of mental health as also suggested by two randomized placebo-controlled trials of antidepressants in Alzheimer's caregivers and PTSD patients [29], [30]. Interestingly, the association was only significant with current anxiety disorders (especially GAD), comorbid with mood disorder or not, but not with mood disorder without anxiety. No previous studies have examined the relationship between resilience and depressive and anxious symptomatology simultaneously. In a prospective bereavement study, Bonnano observed a large resilient group with a relatively healthy mental profile prior to the loss, but also a small group of resilient participants who were highly depressed before bereavement suggesting that numerous pathways to resilience may exist, independently of depression. Unfortunately, anxiety was not examined in this study [1]. The capacity to tolerate high levels of fear and still perform efficiently within a military context has been associated with resilience [31] suggesting that low anxiety trait is associated with resilience. High trait anxiety was also associated with low hardiness in healthy male participants [32]. Our data indicate that in women current anxiety disorder is negatively associated with resilience independently of mood disorder comorbidity. As in other studies we cannot exclude that experiencing current anxiety symptoms may lead persons to perceive themselves as less resilient. This possibility remains to be clarified, especially in men who are less prone to rumination or anxiety disorder [33], [34].

### Resilience and lifetime trauma exposure

The other finding of a positive association between lifetime traumatic exposure and CD-RISC resilience scores is unexpected if we refer to some studies in which childhood abuse was shown to be associated with lower resilience level in adults [35], [36]. However we have already reported that traumatic events could have negative or positive impact on late-life mental health and suicidal ideation [37], [38] and this may depend on the nature of the trauma [39]. Interestingly in our sample, the women having recently experienced the personal trauma of cancer disease were equally distributed among resilience tertiles as those without trauma. By contrast, the women having been exposed during the life to the sudden unexpected death of a close one (*i.e.* a non personal trauma) were mainly in the high resilience tertile (46.8%) with only 11.4% in the lowest tertile. One explanation could be that the exposure to a lifetime trauma and the nature of the trauma may modify the self-evaluation of resilience for women faced with current stressful events, possibly because one's perception of stress is different according to whether a person was exposed or not to a trauma, and the degree of exposition. Other possibilities could involve the recentness of the event “breast cancer” [Mean (SD)  =  24.8 months (8.4)] compared to other traumatic events [Mean (SD)  =  273.6 months (206.4)] or age and social support system at the time of the trauma which was not examined in this study.

Recently Seery et al. have studied the impact of cumulative lifetime adversity on vulnerability and resilience in a longitudinal study [40]. Consistent with prior research on the impact of adversity, they observed linear effects between more lifetime adversity and higher global distress, functional impairment as well as lower life satisfaction. However, they also showed that results yielded quadratic, U-shaped patterns, demonstrating a more complex relationship between lifetime adversity and outcomes than previously supposed. Indeed people with a history of some lifetime adversity (low adversity group) reported better mental health and well-being outcomes than not only people with a high history of adversity but also than people with no history of adversity. Actually people with some prior lifetime adversity were the least affected by recent adverse events. [40]. In our study despite different outcome measures (e.g. “resilience” versus “life satisfaction”) our data may thus suggest that women with intermediary (R2) and higher (R3) resilience scores could correspond to the low adversity group, whereas the high adversity group would be not represented in our sample. Since all the lifetime serious events have not been exhaustively collected in this study, we cannot confirm this hypothesis.

The observation of higher global distress and lower life satisfaction in people with no history of adversity compared to people with history of low adversity [40] could relate to the concept of “posttraumatic growth or adversarial growth” which has been reported following a number of traumatic events, *e.g.* accidents, disasters, cancer, and sexual [41], [42]. Adversarial growth refers to when the process of coping with adversity leads to higher levels of psychological functioning and well-being than previously experienced. This concept includes several dimensions, *e.g.* an enhancement of the relationships with relatives, a change of the views of oneself (for example a greater sense of personal resilience) and in life philosophy [41], [42]. Being confronted with traumatic event may elicit a reevaluation of life goals and priorities, such that individuals emerge with a greater investment in and appreciation of life, interpersonal relationships, spirituality, and personal resources.

It is conceivable that in our study, the measure of current resilience captures both effects, past growth following adversity and “pure” resilience. The fact that CD-RISC scores change in (non-resilient) chronic PTSD patients following pharmacological treatment, suggests that this scale can evaluate resilience but also recovery abilities [28], [30]. Future longitudinal studies in different samples focusing on the effect of trauma exposure on resilience evaluation and growth following adversity are required to explore this question.

Our study has been conducted on a particularly interesting sample with approximately the same proportion of women having directly experienced a trauma (personal trauma, predominantly cancer disease) and women having learned a deadly trauma of a relative (non personal trauma). The average resilience score of the whole sample as measured with the CD-RISC-10 [median (Q25–Q75)  = 27.0 (22–32)] appears lower than that described in a large US population survey [mean (SD)  = 31.8 (5.4)] [35] but similar to that described in a Spanish sample of young adults [mean (SD)  = 27.4 (6.4)] [12]. The difference in the resilience score could be due to the nature of the trauma and to gender, women having lower resilience levels [12], [35], [43]. No significant association was found between cancer history and high resilience level whereas lifetime trauma was strongly and significantly associated with high resilience level (OR = 3.18, p = 0.004). This suggests that this association was more likely related to the traumatic situation surrounding breast cancer (reported by 24.6% of breast cancer survivors) rather than breast cancer itself. A positive association was however still between “history of breast cancer” and the group of intermediate resilience level, compared to low resilience level. As women with a breast cancer history have been described to report high growth in response to adversity [44], [45], this may suggest that this dimension could be more predominant on the resilience measure in the intermediate than in the high resilience level group.

### Limitations and strengths

Some limitations concern survival and self-reported covariates with eventual subsequent recall bias, particularly for childhood trauma. Indeed traumatic life events were gathered using the Watson's PTSD Interview and the traumatic history was not weighted according to number or age at the trauma. As in any observational study the retrospective collection of lifetime traumas precludes definitive conclusion about causation.

This study was conducted on a particular sample (adult women with specific traumatism, 80% of the traumatized women having declared either a cancer disease, either the sudden, unexpected death of a close one as traumatic event) which limits the possibility to generalize to other traumatic events and other population.

The strengths of this study relates to the lifetime mood and anxiety diagnosis using validated instruments including PTSD diagnosis which provides a continuous measure of severity and thus of partial PTSD and the possibility to compare the impact of personal and non-personal trauma. Furthermore, analyses were adjusted for psychoactive treatment and socio-demographic characteristics.

In conclusion, our results stress the need to take into account current anxiety disorder and the nature of lifetime traumas of adulthood in resilience studies. Prospective studies in different samples especially men are required to further specify the determinants of resilience measured with CD-RISC and its association with other positive psychological constructs.
